# Thyroid Hormone and Tissue Repair: New Tricks for an Old Hormone?

**DOI:** 10.1155/2013/312104

**Published:** 2013-02-25

**Authors:** Iordanis Mourouzis, Efstathia Politi, Constantinos Pantos

**Affiliations:** Department of Pharmacology, University of Athens, 75 Mikras Asias Avenue, Goudi, 11527 Athens, Greece

## Abstract

Although the role of thyroid hormone during embryonic development has long been recognized, its role later in adult life remains largely unknown. However, several lines of evidence show that thyroid hormone is crucial to the response to stress and to poststress recovery and repair. Along this line, TH administration in almost every tissue resulted in tissue repair after various injuries including ischemia, chemical insults, induction of inflammation, or exposure to radiation. This novel action may be of therapeutic relevance, and thyroid hormone may constitute a paradigm for pharmacologic-induced tissue repair/regeneration.

## 1. Introduction 

Although the role of thyroid hormone (TH) during development has long been recognized, its role later in adult life remains largely unknown [1]. A growing body of evidence reveals that thyroid hormone may be a major player for the response to stress and its presence crucial to poststress adaptation and recovery. Thus, thyroid hormone is now thought to have a reparative action later in adult life, and this has been recently documented in several studies; see Table 1.

## 2. Adaptation to Environmental Stress and Species Evolution: The Critical Role of Thyroid Hormone

The most important challenge that living organisms faced during species evolution was the ability to adapt to the transition from the aquatic environment, a condition of low oxygen, to the ground, an oxygen-rich state. This required a gene programming that would enable organ protection and remodeling during this transition. Interestingly, studies on amphibians revealed that thyroid-hormone-regulated gene programming is critical for the metamorphosis of tadpoles into juvenile frogs [2]. Several studies have shown that the morphological and functional changes of metamorphosis are the result of alterations in the transcription of specific sets of genes induced by TH and TH alterations can lead to developmental failures [3–6]. 

## 3. Thyroid Hormone and Stress Response: An Evolutionary Conserved Mechanism 

**Figure 1:**
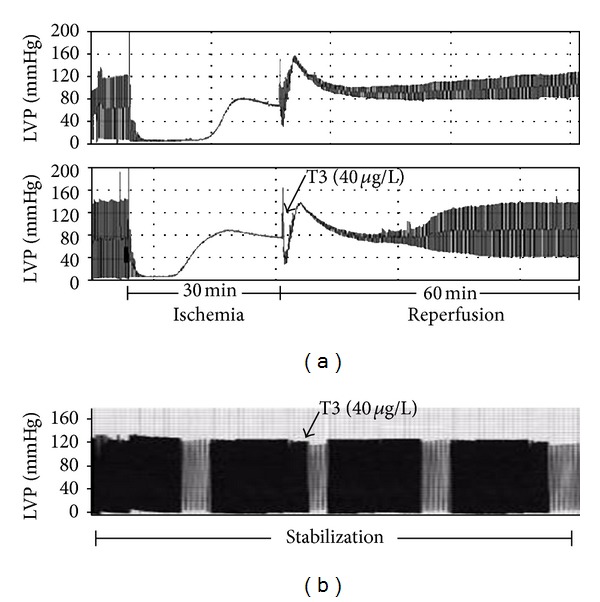
Langendorff recordings of left ventricular pressure (LVP) from isolated rat hearts subjected to zero-flow global ischemia followed by reperfusion (a) and hearts subjected only to stabilization (b). Triiodothyronine (T3) administration at reperfusion improves postischemic recovery of function, whereas T3 during stabilization does not affect contractile function.

The potential role of thyroid hormone in stress response has been, until now, underestimated. However, thyroid hormone signaling is altered during various stressful stimuli and thyroid hormone is crucial to poststress recovery and injury repair [7–9]. Interestingly, the importance of thyroid hormone for stress response has been documented in several species ranging from fish to humans [10]. Thus, exposure of air-breathing perch to water-born kerosene resulted in low T3 and unfavorable metabolic changes, while the administration of TH reversed this response [11]. Along this line, cold stunning Kemp's ridley sea turtles had undetectable levels of thyroid hormone, and recovery was observed only in those who recovered thyroid hormone levels in blood [12]. Interestingly, a similar response is also observed in humans. In fact, after an index event, such as myocardial infarction, T3 levels significantly drop and lower levels of T3 are associated with high mortality [13, 14]. Furthermore, T3 levels are strongly correlated to early and late recovery of cardiac function, with T3 levels at 6 months to be an independent predictor of the recovery of the myocardium [15]. In fact, patients who spontaneously recover T3 levels in plasma after myocardial infarction are those with markedly improved cardiac functional recovery [15]. These observations provide clear evidence that thyroid-hormone-regulated mechanisms may be evolutionary conserved and are crucial to the response to stress and poststress recovery and tissue repair [11]. Along this line, several studies have demonstrated the reparative action of thyroid hormone. We have recently shown that T3 at a dose which had no effect on noninjured myocardium significantly limited apoptosis in the ischemic myocardium and improved postischemic function in an isolated rat heart model of ischemia-reperfusion. This effect was due to the suppression of the ischemia-reperfusion-induced activation of the proapoptotic p38 MAPK [16, 17] as shown in, Figure 1.

## 4. Thyroid Hormone: The “Black Box of Repair?”

Accumulating experimental evidence shows that thyroid hormone may play a critical role in the repair after injury in almost every tissue and organ as shown in Table 1. This probably implies that organisms may have a common mechanism of repair which may be regulated by thyroid hormone and has been established during evolution. Thus, thyroid hormone was shown to control DNA repair after irradiation-induced damage in mouse intestine [38]. A single dose of T3 in rats significantly diminished hepatocellular injury induced by ischemia-reperfusion (I/R) when given 48 h before the I/R protocol. This effect was mediated by a T3 transient oxidative stress, and thus, it was abrogated by the administration of antioxidant N-acetyl-cysteine [23]. Thyroxine was cytoprotective in toxic and ischemic injury in kidney [24, 26]. Thus, T3 administration 24 h prior to renal ischemia could precondition against ischemia-reperfusion (I/R) injury. This was evident by a marked decrease in I/R-induced proteinuria. T3 treatment also improved lipid peroxidation biomarkers and increased antioxidant enzymes [24]. In another study, T4 administration immediately or 24 h after ischemia resulted in higher Inulin clearance and preserved cellular integrity [26]. In accordance with these observations in animal models, T4 was shown to be cytoprotective, in a cellular model of reoxygenation injury in isolated proximal tubule cells [25]. Such evidence may provide an explanation to the clinical observation that low T3 has been associated with increased mortality in hemodialyzed patients [39]. T3 treatment prevented streptozocin-induced toxic injury in pancreatic cells. This effect was associated with an increased activation of the prosurvival Akt signaling [27]. Similarly, T3 was shown to improve function and survival of rat pancreatic islets in in vitro cell cultures [40]. Moreover, T3 was found to preserve ovarian granulose cells exposed to paclitaxel. In fact, T3 significantly reduced the paclitaxel-induced cell injury via downregulation of caspase3 and Bax and upregulation of Bcl2 [28]. T3 pretreatment in rats instilled with an isosmolar 5% albumin solution resulted in the upregulation of alveolar epithelial fluid clearance [41]. T3 was also shown not only to stimulate alveolar fluid clearance in normal but also in hypoxia-injured lungs [29]. The administration of T3 attenuated neointimal formation after balloon injury of carotid artery [35]. Thyroid hormone enhanced transected axonal regeneration and muscle reinnervation following rat sciatic nerve injury [22] and improved recovery of sensory function [21]. Similarly, thyroid hormone was shown to be essential for muscle regeneration after injury [33, 34]. Thyroid hormone promoted the survival of injured neurons [18] and enhanced remyelination in demyelinating inflammatory disease [20]. Thyroid hormone has also been shown to accelerate wound healing in mice and guinea pigs [36, 37].

## 5. Conclusions 

Thyroid hormone appears to be a common player for the organ development and response to stress. Thyroid hormone was crucial for species evolution, and thyroid-hormone-regulated mechanisms have been evolutionary conserved and play an important role early during development. However, recent research has revealed that thyroid hormone has a reparative role later in adult life. This novel action may be of therapeutic relevance, and thyroid hormone may constitute a paradigm for pharmacologic induced tissue repair/regeneration.

## Figures and Tables

**Table 1:** Accumulating experimental evidence shows that thyroid hormone may play a critical role for the repair after injury in several tissues and organs.

Study	Type of treatment	Tissue	Type of injury	Outcome
Shulga et al. 2009 [18]	Treatment with T4 after injury	Mouse hippocambal slices	Mechanical injury	Increased number of neurons, reduced caspase-3 activation, and increased axonal regeneration
Hiroi et al. 2006 [19]	Treatment with T4 after ischemia	Mouse central nervous system	Transient focal ischemia	Reduced cerebral infarct volume, and improved neurological deficit score
Fernandez et al. 2004 [20]	Treatment with T4 after injury	Rat nervous system	Chronic demyelinating inflammatory disease	Enhancement of remyelination
Papakostas et al. 2009 [21]	Treatment with T3 after injury	Rat sciatic nerve	Nerve transection	Increased recovery of sensory function
Panaite and Barackat-Walter 2010 [22]	Treatment with T3 after injury	Rat sciatic nerve	Nerve transection	Increased number of regenerated axons, improved muscle reinnervation
Fernández et al. 2007 [23]	Pretreatment with T3	Rat Liver	Ischemia-reperfusion	Reduced injury (serum AST and ALT levels)
Ferreyra et al. 2009 [24]	Pretreatment with T3	Rat kidney	Ischemia-reperfusion	Reduced proteinuria
Erkan et al. 2003 [25]	Pretreatment with T4	Rabbit proximal tubule cells	Anoxia reoxygenation	Better preservation of cellular structure
Sutter et al. 1988 [26]	Treatment with T4 after ischemia	Rat kidney	Ischemia-reperfusion	Improved kidney function, preserved cellular morphology
Verga Falzacappa et al. 2011 [27]	Contemporary T3 treatment	Mouse pancreas	Streptozocin-induced toxicity	Increased number, shape, and dimension of islets, increased insulin and glucagon levels
Verga Falzacappa et al. 2012 [28]	Contemporary T3 treatment	Rat ovarian granulosa cells	Chemotherapy induced toxicity	Increased number of survived cells, reduced apoptosis
Bhargava et al. 2008 [29]	Pretreatment with T3	Rat lung	Hyperoxia injury	Increased alveolar fluid clearance
Pantos et al. 2011 [16]	Treatment with T3 after ischemia	Rat heart	Ischemia-reperfusion	Increased recovery of function, reduced injury and apoptosis
Pantos et al. 2009 [17]	Treatment with T3 after ischemia	Rat heart	Ischemia-reperfusion	Increased recovery of function, reduced injury
Pantos et al. 2002 [30]	Pretreatment with T4	Rat heart	Ischemia-reperfusion	Increased recovery of function
Kuzman et al. 2005 [31]	Pretreatment with T3	Neonatal rat cardiomyocytes	Serum starvation	Increased cell viability, reduced apoptosis
Chen et al. 2008 [32]	Treatment with T3 after infarction	Rat heart	Acute myocardial infarction	Improved LV function, reduced apoptosis
Dentice et al. 2010 [33]	Treatment with T3 after injury	Mouse skeletal muscle	Mechanical injury	Improved muscle regeneration
Marsili et al. 2011 [34]	Induction of D2-increased T3	Mouse skeletal muscle	Skeletal muscle injury	Improved muscle regeneration
Fukuyama et al. 2006 [35]	Treatment with T3 after injury	Rat carotid artery	Mechanical injury	Attenuation of VSMC proliferation and neointimal formation
Safer et al. 2004 [36]	Treatment with T3 after injury	Mouse skin	Wound	Accelerated wound healing, increased keratinocyte proliferation
Kassem et al. 2012 [37]	Local T3 treatment	Guinea pig skin	Wound	Reduction in the wound surface area
